# Glyco-engineered Long Acting FGF21 Variant with Optimal Pharmaceutical and Pharmacokinetic Properties to Enable Weekly to Twice Monthly Subcutaneous Dosing

**DOI:** 10.1038/s41598-018-22456-w

**Published:** 2018-03-09

**Authors:** Yan Weng, Tetsuya Ishino, Annette Sievers, Saswata Talukdar, Jeffrey R. Chabot, Amy Tam, Weili Duan, Kelvin Kerns, Eric Sousa, Tao He, Alison Logan, Darwin Lee, Dongmei Li, Yingjiang Zhou, Barbara Bernardo, Alison Joyce, Mania Kavosi, Denise M. O’Hara, Tracey Clark, Jie Guo, Craig Giragossian, Mark Stahl, Roberto A. Calle, Ron Kriz, Will Somers, Laura Lin

**Affiliations:** 1 BioMedicine Design, Pfizer Worldwide Research and Development, 610 Main Street, Cambridge, MA 02139 USA; 2 BioMedicine Design, Pfizer Worldwide Research and Development, 1 Burtt Road, Andover, MA 01810 USA; 3 BioMedicine Design, Pfizer Worldwide Research and Development, 558 Eastern Point Road, Groton, CT 06340 USA; 4 BioMedicine Design, Pfizer Worldwide Research and Development, 10770 Science Center Drive, San Diego, CA 92121 USA; 5 Internal Medicine, Pfizer Worldwide Research and Development, 1 Portland Street, Cambridge, MA 02139 USA; 6 Internal Medicine, Pfizer Worldwide Research and Development, 558 Eastern Point Road, Groton, CT 06340 USA

## Abstract

Pharmacological administration of FGF21 analogues has shown robust body weight reduction and lipid profile improvement in both dysmetabolic animal models and metabolic disease patients. Here we report the design, optimization, and characterization of a long acting glyco-variant of FGF21. Using a combination of N-glycan engineering for enhanced protease resistance and improved solubility, Fc fusion for further half-life extension, and a single point mutation for improving manufacturability in Chinese Hamster Ovary cells, we created a novel FGF21 analogue, Fc-FGF21[R19V][N171] or PF-06645849, with substantially improved solubility and stability profile that is compatible with subcutaneous (SC) administration. In particular, it showed a low systemic clearance (0.243 mL/hr/kg) and long terminal half-life (~200 hours for intact protein) in cynomolgus monkeys that approaches those of monoclonal antibodies. Furthermore, the superior PK properties translated into robust improvement in glucose tolerance and the effects lasted 14 days post single SC dose in ob/ob mice. PF-06645849 also caused greater body weight loss in DIO mice at lower and less frequent SC doses, compared to previous FGF21 analogue PF-05231023. In summary, the overall PK/PD and pharmaceutical profile of PF-06645849 offers great potential for development as weekly to twice-monthly SC administered therapeutic for chronic treatment of metabolic diseases.

## Introduction

Fibroblast growth factor 21 (FGF21) is a fasting-induced hormone originally identified as a protein that increased glucose uptake in adipocytes^1^. It is primarily a hepatokine expressed in pancreas, skeletal muscle, and brown adipose tissue^2^, signaling as a metabolic regulator through FGF receptor 1c isoform (FGFR1c) and co-receptor beta-klotho (KLB)^3–6^. Pharmacological administration of FGF21 in preclinical obese and/or diabetic animal models have shown improved insulin sensitivity and glucose tolerance, increased energy expenditure, enhanced fat utilization, reduced hepatic and serum triglyceride level, and caused weight loss^1,3,7–9^. These have led to the establishment of FGF21 as an attractive therapeutic target for the treatment of metabolic diseases^10,11^.

Wild type (WT) FGF21 is a 19 kDa protein with *in vivo* half-life of ~0.5–2 hours. This short half-life represents a substantial challenge for development as a therapeutic for chronic treatment of metabolic diseases as it would require daily injections^12,13^. In addition, the poor physicochemical properties of the native protein including low intrinsic stability, serum degradation, and high aggregation propensity, presents additional challenges during development and manufacturing, particularly for subcutaneous (SC) administration at therapeutic doses^14,15^. Several approaches to improve the pharmaceutical properties and pharmacokinetics (PK) of FGF21 have been described, including Fc fusion, polymer conjugation, antibody conjugation for half-life extension, introduction of disulfide bonds to increase manufacturing stability, and site directed mutagenesis to improve protease stability^15–18^. To date, two FGF21 analogues have been tested in humans, and caused body weight loss and improved lipid profile. While both molecules showed improved pharmaceutical properties compared to WT FGF21, one still required daily SC dosing as a result of a short half-life, and the other required twice weekly intravenous (IV) dosing as a result of poor SC bioavailability and proteolytic instability within the C-terminus of FGF21^12,19–21^.

One potential approach to improve physicochemical properties and simultaneously reduce proteolysis is glyco-engineering. N-linked glycan engineering has been used successfully to extend the circulating half-life of recombinant human erythropoietin (huEPO) via a sialic acid containing glycan dependent mechanism^22^. In addition, presence of glycans can lead to increased solubility and stability by shielding the hydrophobic patches of the protein with highly soluble, charged sugar moieties. Furthermore, sequence mutations introduced to enable glycan engineering would be masked by the glycan itself, thereby minimizing the risk of being recognized by the host immune system^23^. A hyperglycosylated analogue of huEPO was successfully developed and marketed as Aranesp^®^ that has a threefold increase in serum circulating half-life, allowing for less frequent dosing in patients compared to rhEPO^24^. Here we extended this methodology to FGF21 as part of a combination approach to improve its properties. We report the design, optimization, and characterization of a long acting glyco-variant of FGF21, PF-06645849, with a PK profile that allows for once weekly to bi-weekly dosing, and a pharmaceutical profile that supports SC administration.

## Results

### Glycoengineering for improved serum stability

Proteolytic cleavage of FGF21 occurs rapidly *in vitro* and *in vivo*^15,19,20^ (Supplemental Fig. S1a), In particular, it is known that the cleavage between residues Pro171 and Ser172 leads to substantial loss of function^25–27^. Given the volume that a single sialylated, complex carbohydrate can occupy (approximately 2542 Å^3^)^28^, we hypothesized that the addition of an appropriately positioned carbohydrate moiety on native FGF21 would prevent cleavage by serum proteases through steric hindrance. We set out to design glyco-variants that will prevent serum proteolysis while maintaining biological activity. We applied an N-glycan scan with the following design principles for introducing N-glycosylation mutations (Asn-X-Ser/Thr). First, using a homology model built from FGF19 crystal structure (PDB 2P23) (Supplemental Fig. S1b), we identified residues located on potentially unstructured regions of FGF21 covering 15 sites in the N-terminus, 3 sites in the middle, and 25 sites in the C-terminus of FGF21. Second, since N-glycosylation efficiency can be greatly reduced by proline residue at either position of X or Y in the consensus sequence of Asn-X-Ser/Thr-Y, proline was replaced with glycine whenever it was found at the positions X or Y. Additionally, because aspartate and glutamate residues at the position X can be strong inhibitors for efficient N-glycosylation, they were replaced with glycine. Lastly, when residue(s) at position +2 needed to be mutated, we chose threonine over serine because it has been shown that threonine at that position tends to produce higher N-glycosylation occupancy than serine^29^. A total of 43 positions were selected for N-glycosylation scan (Supplemental Fig. S1c,d and e).

**Figure 1:**
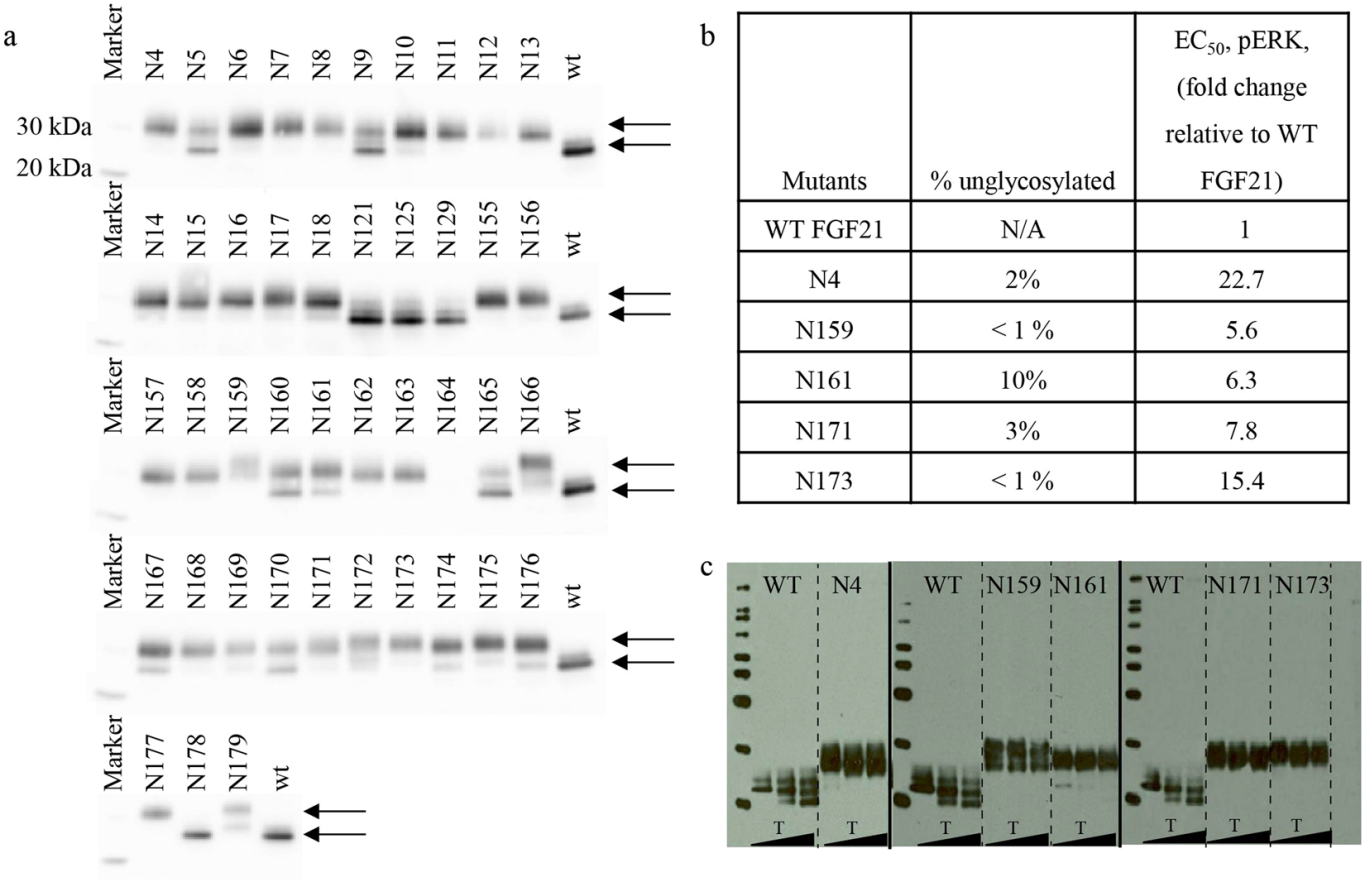
Characterization of selected glyco-engineered FGF21 variants. (**a**) Western blot analysis of glyco-engineered FGF21 variants. Lower and upper arrows are shown for each blot to indicate unglycosylated WT FGF21 and glycosylated proteins, respectively. (**b**) Percent of unglycosylated protein (determined by Caliper LabChip capillary gel electrophoresis) and pERK activity in hASC for selected glyco-variants. (**c**) Western blot analysis of serum stability of selected FGF21 variant following incubation in human serum for up to 96 hr (solid dividing lines indicate separate blots; dash line indicates different proteins on the same blot).

Individual N-glycosylation mutants were transiently expressed in HEK293F cells. Conditioned media was subjected to SDS-PAGE under reducing condition, and western-blot analysis was used to assess the expression level and efficiency of N-glycosylation. It appeared that the N-glycosylated variants migrated with mobility corresponding to molecular weight of approximately 30 kDa whereas the non-glycosylated variants migrated at approximately 26 kDa (Fig. 1a). A total of 30 variants showed efficient N-glycosylation (Fig. 1a), while the rest of variants showed heterogeneous and incomplete incorporation of N-glycosylation (Supplemental Table S1).

The *in vitro* potency of these N-glycosylation variants was evaluated using a previously published pERK assay in hASC^25^. Of note, many of the engineered glyco-variants showed considerable EC_50_ reduction regardless of their N- or C-terminal locations (Supplemental Table S1), indicative of a modest to substantial impact on binding to the FGFR1c/KLB complex in the presence of a bulky glycan. Based on the glycosylation efficiency and biological activity in pERK assay, we chose five N-glycosylation variants, denoted as N4 (FGF21[P4N/D5G]), N159 (FGF21[D159N/G161T]), N161 (FGF21[G161N]), N171 (FGF21[P171N/Q173T]) and N173 (FGF21[Q173N/R175T]) for further profiling (Fig. 1b).

Next, the impact of these N-glycosylation sites on the proteolysis of FGF21 was examined in a human serum assay. N161, N171 and N173 appear to be stable in human serum for up to 96 hours (indicated as no appearance of degraded products over time); in contrast, degradation of WT FGF21 was clearly indicated as appearance of increasing amount of lower MW species over time (Fig. 1c & Supplemental Fig. S2 for full blot images). Of these three variants, N171 demonstrated the best retention of potency and the highest N-glycan occupancy (Fig. 1b). Given this is also the previously identified site of proteolysis in human serum (Supplemental Fig. S1a), N171 was chosen for further optimization.

### Fc-fusion for half-life extension

Feasibility of an Fc-fusion approach to extend the *in vivo* half-life of FGF21 [N171] was investigated. It was noted that, compared to native FGF21, human IgG1 Fc fused at the C-terminus of FGF21 (WT FGF21-Fc) caused substantial loss in potency compared to format where Fc was fused at the N-terminus of FGF21 (Fc-WT FGF21) (Supplemental Fig. S3). A similar finding was reported by Hecht, *et al*.^15^. Consequently, Fc-FGF21 fusion format was chosen as the framework for further engineering.

**Figure 2:**
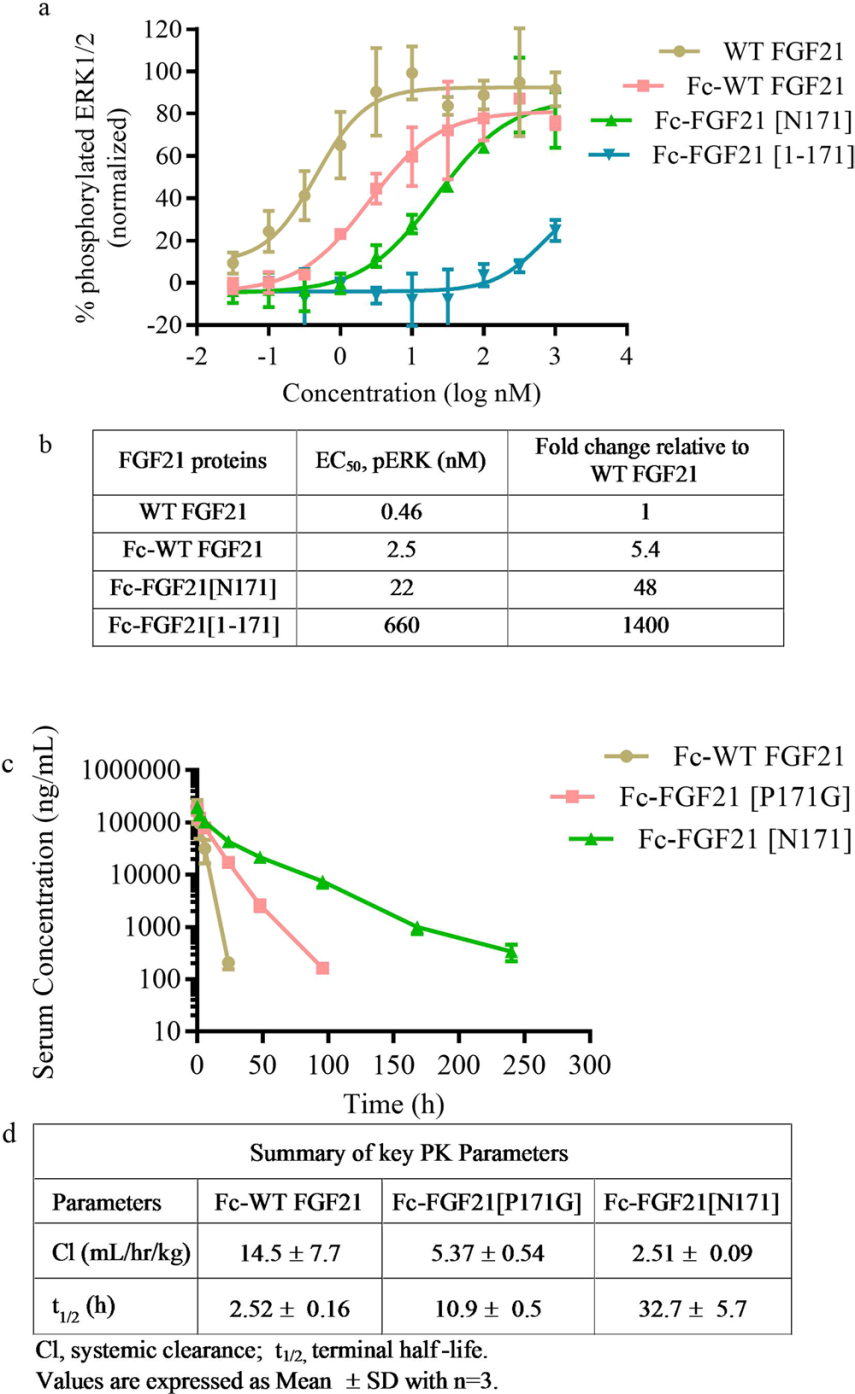
Characterization of HEK transient cell line-expressed Fc-FGF21 proteins. (**a**) pERK phosphorylation assay of WT FGF21, Fc-WT FGF21, Fc-FGF21[N171], and Fc-FGF21[1–171]. Values are Mean ± SD (n = 3). (**b**) Summary of EC_50_ of Fc-FGF21 variants in pERK assay. (**c**) Mean serum concentration-time profiles following a single IV dosing of Fc-WT FGF21, Fc-FGF21[P171G], and Fc-FGF21[N171] at 10 mg/kg. Values presented are Mean ± SD (n = 3). (**d**) Summary of key PK parameters. Values presented are Mean ± SD (n = 3).

To better understand the overall impact of an N171 mutation in the context of a Fc-fusion protein, we generated a set of benchmark molecules, Fc-FGF21[1–171] (the C-terminal truncated version that was shown to be largely inactive^26,27^) and Fc-FGF21[P171G] (a previously identified long-acting analogue^15,30^), for head-to-head comparison. Compared to Fc-WT FGF21, we observed ~260x loss in potency for Fc-FGF21[1–171], whereas only ~9x loss of potency for Fc-FGF21[N171]. Of note, the potency shift between Fc-WT FGF21 and Fc-FGF21[N171] (Fig. 2a,b) was similar to that between FGF21[N171] and WT FGF21 (Fig. 1b).

Based on the lack of turnover of FGF21[N171] in the serum stability assay (up to 96 hr) (Fig. 1b), the half-life extending effect of these Fc-FGF21 variants were evaluated in rats following a single IV administration at 10 mg/kg. As shown in Fig. 2c,d, IV PK of Fc-FGF21[N171] is superior to the other two, with an *in vivo* half-life ~13x and 3x longer, and clearance ~6x and 2x lower than those of Fc-WT FGF21 and Fc-FGF21[P171G], respectively. The Fc proteins used in this study were transiently expressed using HEK293F cells. In the case of Fc-FGF21[N171], the engineered glycan in HEK transient material was heterogeneous and with noticeable amount of high mannose (results not shown) that could in theory negatively impact the *in vivo* clearance^31^. We expected that CHO cell-derived proteins with normal processing and sialyation on the engineered glycan would result in further improvement of the pharmacokinetic properties of Fc-FGF21[N171].

### Protein engineering for manufacturability in CHO stable cell line

**Figure 3:**
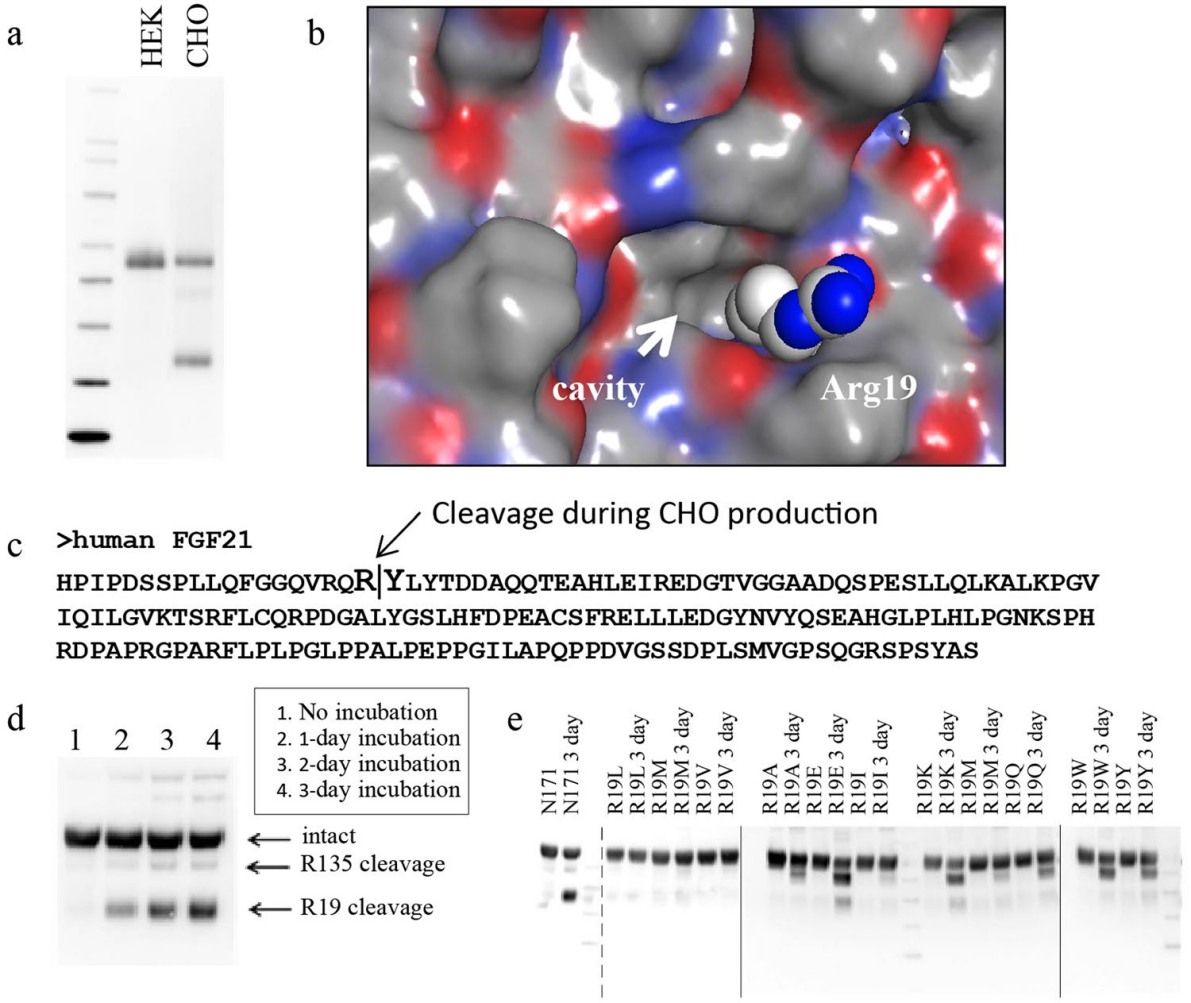
Protein engineering to enable manufacturing in CHO stable cell lines. (**a**) Western blot analysis of HEK- and CHO-produced Fc-FGF21[N171] proteins; (**b**) MOE model of the Arg19 protease susceptible site. (**c**) Sequence of human FGF21. (**d**) Western blot analysis of HEK-produced Fc-FGF21[N171] following incubation in conditioned CHO cell media. (**e**) Western blot analysis of HEK-produced R19 mutants of Fc-FGF21[N171] following incubation in conditioned CHO cell media for 3 days; Solid line indicates separate blots; dashed line indicates two duplicate lanes were cropped out.

Next, we tested the ability to produce Fc-FGF21 [N171] in our standard CHO stable cell line used for large-scale production. Surprisingly, we observed a major cleaved product of Fc-FGF21[N171] that is specific to stable CHO cell production, but not in previous transient HEK cell productions (Fig. 3a). The cleavage site was identified using mass spectrometry as between Arg19 and Tyr20 (numbering as WT FGF21) in Fc-FGF21[N171] (Fig. 3c). We hypothesized that the cleavage was mediated by secreted proteases accumulated during CHO cell culture. To prove this, HEK-produced intact Fc-FGF21[N171] was incubated in conditioned media (CM) following CHO cell culture for 7 days. Consistent with our hypothesis, we observed time-dependent appearance of the R19 cleavage product that recapitulated the cleavage pattern of the CHO-derived Fc-FGF21[N171] (Fig. 3d).

Based on FGF21 structure model, Arg19 is the first structured residue from the N-terminus and appears to be located in a hydrophobic pocket (Fig. 3b). Early glycan engineering scan indicated that this region does not tolerate the addition of an engineered glycan (Supplemental Table S1, N16-N18); therefore, we decided to explore single point mutations to overcome this proteolysis issue in CHO cell. Since the Cβ atom of the arginine residue is completely buried in the hydrophobic environment, we hypothesized that a small, hydrophilic residue such as Gly, Ser, Thr, Asp, Asn, His and Cys as well as Pro would destabilize the local structure. Hence, these residues were excluded for the replacement of Arg19. Based on these analyses, Arg19 was replaced with Lys, Ala, Gln, Glu, Met, Ile, Val, Leu, Trp, Phe, and Tyr in the context of Fc-FGF21[N171]. These variants were transiently expressed in HEK293F cells, purified, and tested in the CHO CM based proteolysis assay described above. The results showed that replacement of R19 with hydrophobic residues with aliphatic side chain such as Met, Leu, Ile and Val completely prevented the Arg19-Try20 cleavage (Fig. 3e and Supplemental Fig. S4 for full blot images). In addition, except for R19E, all the other mutations showed minimal impact on *in vitro* potency (Supplemental Table S2).

**Figure 4:**
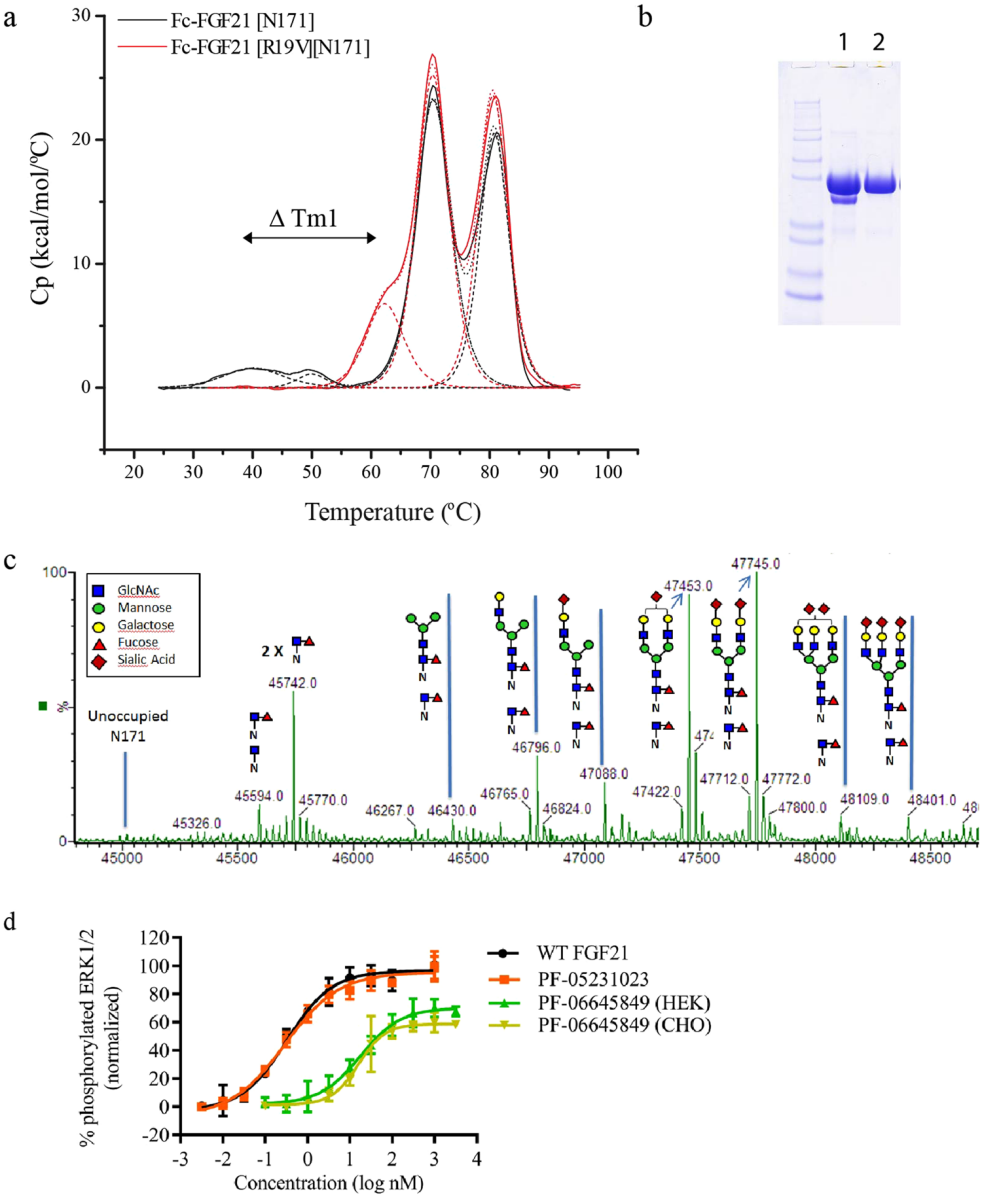
Analytical characterization of PF-06645849. (**a**) DSC traces of Fc-FGF21 [N171] (solid black line) and Fc-FGF21 [N171][R19V] (solid red line) in PBS. Mid-point transition temperature fits are shown in dashed lines. (**b**) Western blot analysis of purified PF-06645849 produced in CHO cell lines. Lane 1, post ProteinA elution; lane 2, post ButylFF (GE) flow-through step. (**c**) Mass spec glycan analysis of PF-06645849 after removal of Fc sugar using IgGZero enzyme. IgGZero hydrolyzes the β1,4 linkage between the core GlcNAc residues in the Fc-glycan, leaving the innermost GlcNAc intact on the Fc (peak labeled 2x). Remaining Glycan structures derived from [N171] are assigned to the corresponding m/z values. (**d**) pERK phosphorylation assay of WT FGF21, PF-05231023, and HEK- and CHO-produced PF-06645849. Values presented are Mean ± SD (n = 4).

To further rank these variants, we compared the conformational stability of Fc-FGF21[N171] R19 variants Met, Leu, Ile, and Val with the parental molecule using differential scanning calorimetry (DSC). We observed four distinct unfolding transitions for Fc-FGF21[N171] with the mid-point transition temperatures (T_m_) of 40.2, 50.0, 70.5 and 80.8 °C, respectively (Fig. 4a). The transitions at 70.5 and 80.8 °C can typically be attributed to C_H_2 and C_H_3 domains of Fc region^32^, the transitions at 40.2 and 50.0 °C are therefore likely related to thermal transitions of FGF21. In contrast, R19M, R19L, R19I and R19V variants of Fc-FGF21[N171] all yielded a single transition T_m_1 at 50.9, 54.7, 55.4, and 62.3 °C, respectively, for FGF21 domain. In particular, the R19V mutation showed the highest stability with the largest T_m_1 increase of 22 °C compared to that of Fc-FGF21[N171] (Fig. 4a), therefore R19V was prioritized as the lead. *In silico* EpiVax analysis^33^ demonstrated that R19V mutation did not introduce additional predicted T cell binding epitopes.

Upon selection of the R19V mutation in Fc-FGF21[N171], the manufacturability of Fc-FGF21[R19V][N171] in CHO cells was revisited. As shown in Fig. 4b (or Supplemental Fig. S5 for full gel image), the incorporation of R19V successfully eliminated the CHO cell specific degradation event. The protein A-purified material still contained a minor, lower molecular weight component (Fig. 4b, lane 1). This species was identified by mass spec to correspond to a cleavage between residue Arg135 and Phe136 of FGF21 (numbering as WT FGF21). Given the relative low level of this impurity, a hydrophobic interaction chromatography step was developed and used to completely remove this species during scale up production (Fig. 4b, lane 2).

Numerous examples have demonstrated that glycan structure of proteins, and in particular, degree of sialylation, play critical roles in modulating PK and clearance of these molecules^22,31,33,34^. We therefore carried out a more detailed glycan analysis of Fc-FGF21[R19V][N171] produced from stable CHO cells by mass spectrometry. Fc-glycans were selectively removed using enzyme IgGZero leaving behind the inner most GlcNAc allowing the assignment of remaining, N171 derived glycan type by m/z (Fig. 4c). This analysis demonstrated that the engineered glycan at N171 was processed to mature, complex carbohydrates with normal degree of branching and terminal sialylation, similar to those found in circulating native proteins such as EPO^23^. No significant high mannose containing glycan was present (Fig. 4c).

**Figure 5:**
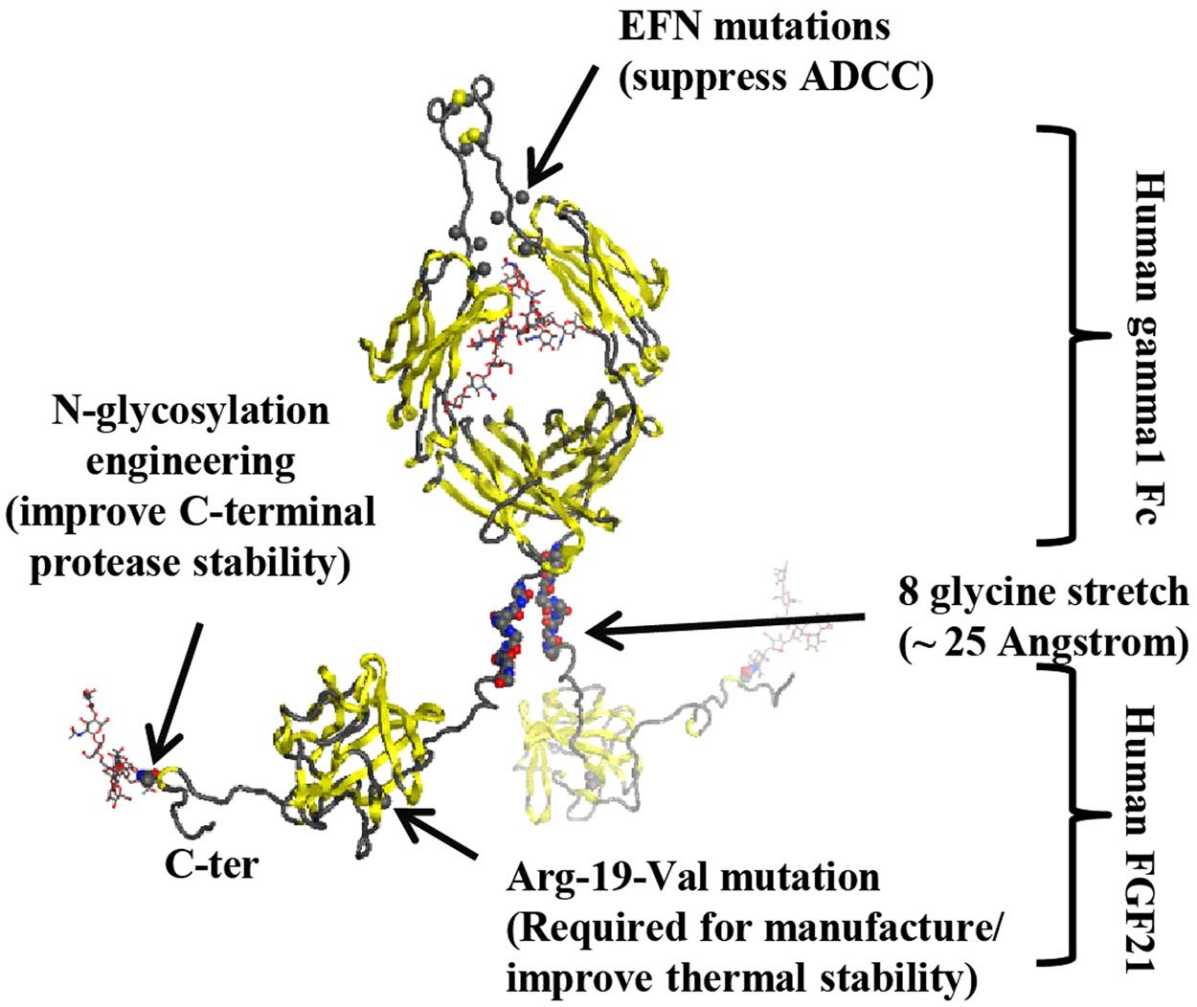
Modeled structure of PF-06645849 depicting the dimeric human IgG1 Fc with a C-terminal FGF21[R19V][N171] fusion via a (Gly)_8_ linker on each chain. Effector function null (EFN) mutations were introduced in the lower hinge region in the Fc C_H_2 domain to ameliorate unwanted antibody-dependent cell-mediated cytotoxicity (ADCC).

The *in vitro* pERK activity of CHO-derived Fc-FGF21[R19V][N171] was shown to be the same as the HEK-derived protein, with EC_50_ of ~16 nM for Fc-FGF21[R19V][N171] (vs. ~0.3 nM for WT FGF21) (Fig. 4d). Additional design features of Fc-FGF21[R19V][N171]included an eight amino acid glycine linker between the Fc and FGF21, and the incorporation of effector function null mutations into the Fc domain^35–37^ to suppress antibody-dependent cell-mediated cytotoxicity (Fig. 5). Taken together, Fc-FGF21[R19V] [N171] was chosen as the lead and was designated as PF-06645849.

### Formulation stability and viscosity

**Figure 6:**
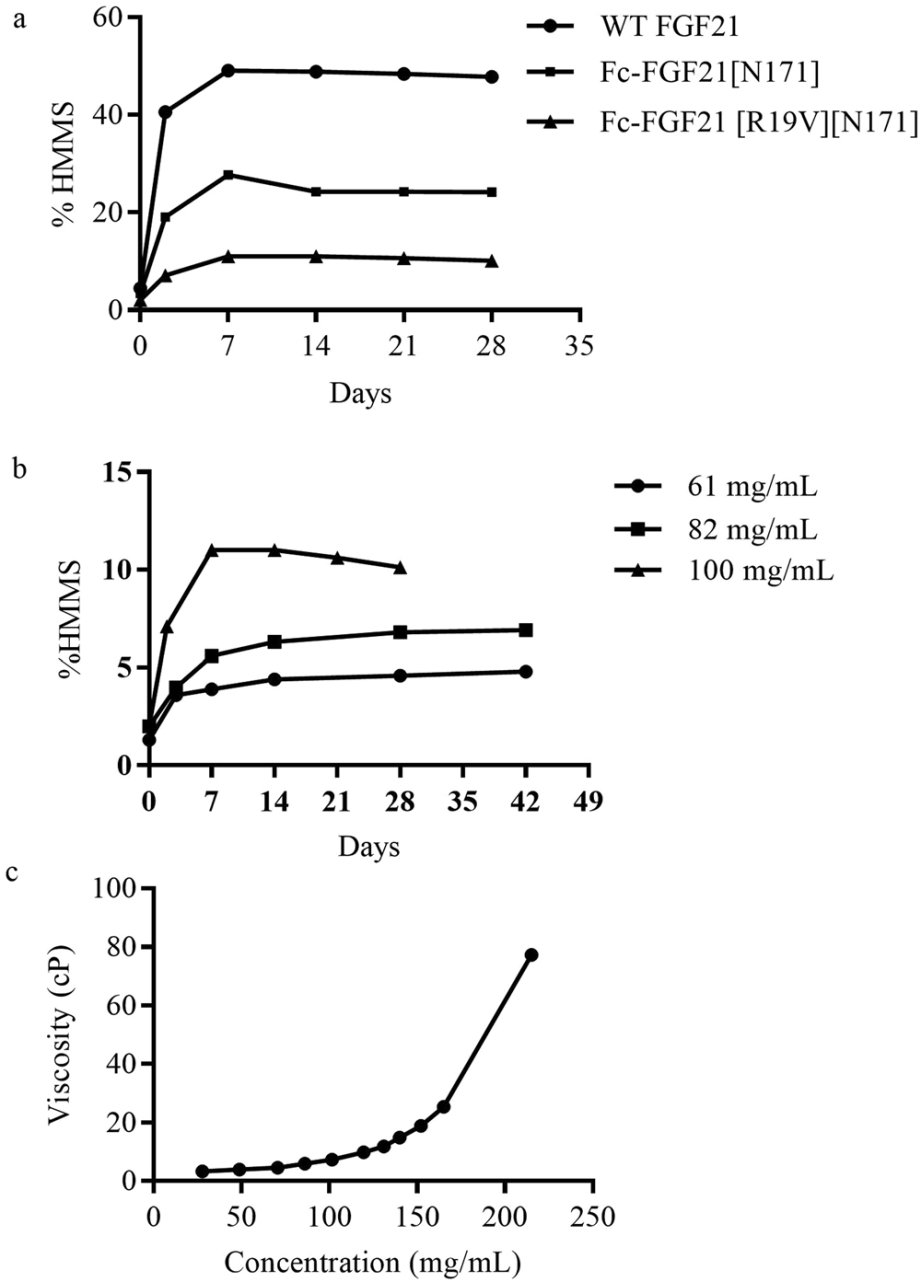
Formulation characterization of PF-06645849. (**a**) % HMMS formation at 25 °C in Tris pH 7.5 formulation for WT FGF21 (40 mg/mL), Fc-FGF21[N171] (100 mg/mL) and PF-06645849/Fc-FGF21 [R19V][N171] (100 mg/mL) over time. (**b**) % HMMS formation for PF-06645849 at pH 7.5/25 °C over time at concentrations of 61, 82, and 100 mg/mL, respectively. (**c**) Viscosity of PF-06645849/Fc-FGF21[R19V][N171] over concentration ranges. All experiments were carried out as n = 1.

We carried out a multi-week high concentration stability assessment of PF-06645849 and Fc-FGF21[N171] at 100 mg/mL in a standard formulation that is compatible with SC injection (20 mM Tris pH 7.5, 8.5% glucose), and used WT FGF21 at 40 mg/ml as an equimolar comparator. Analytical size exclusion chromatography (aSEC) was used to monitor the formation of high molecular mass species (HMMS). Similar to previous report of rapid rates of aggregation in a pH 8.0 formulation for WT FGF21^15^, we also observed high rate of aggregation of the WT FGF21 in the standard formulation (Fig. 6a). In contrast, Fc-FGF21[N171] showed greatly improved stability over WT FGF21, with both the rate and final % HMMS formation reduced in half over the entire study course. PF-06645849 demonstrated a further improvement over Fc-FGF21[N171], the maximum % HMMS at 100 mg/ml only reached a stable level of 11% during the 4 week study. Further analysis showed that the HMMS formation is concentration dependent. At ~60 mg/mL, <5% HMMS formation was observed for PF-06645849 at the end of 6 week study at 25 °C (Fig. 6b). Additional formulation development will likely be able to further improve this high concentration stability profile.

SC administration of biologics can be complicated by the tendency of some proteins to form highly viscous solutions at the concentrations required for efficacy^38^. The backpressure for injection through fine needles becomes prohibitively high above viscosities of 15–20 centipoise (cP). In addition tangential flow filtration steps during manufacturing become more challenging for highly viscous solutions. For these reasons we evaluated the viscosity of PF-06645849. Figure 6c shows that PF-06645849 has low viscosity (<10 cP) up to a concentration of 119 mg/mL in the Tris.HCl pH 7.5 formulation. This viscosity profile is similar to a typical mAb based biotherapeutics with SC dosing regimen^39^, underscoring the substantially improved physicochemical properties of PF-06645849 over previously reported FGF21 analogues used in the clinic.

### PK characterization

**Figure 7:**
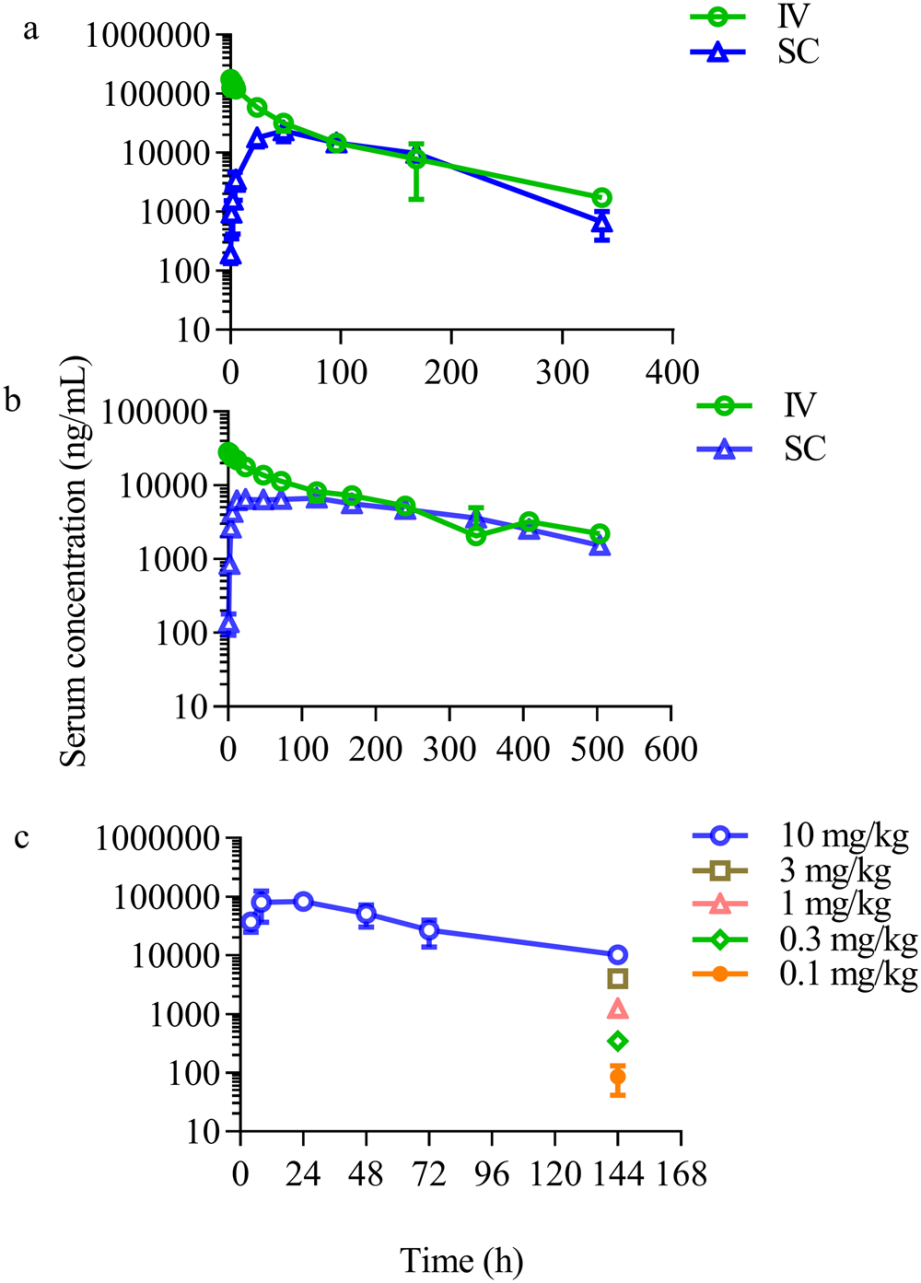
PK profiles of PF-06645849 in rats, monkeys, and mice. (**a**) Mean serum concentration-time profiles in rats following single IV or SC administrations at 10 mg/kg. (**b**) Mean serum concentration-time profiles in monkeys following single IV or SC administration at 1 mg/kg. (**c**) Mean serum concentration-time profiles in ob/ob mice following single SC administration at 10 mg/kg; or serum concentration at 144 hr post single SC dose at 0.1, 0.3, 1, and 3 mg/kg, respectively. Values presented are Mean ± SD with n = 3 for rats and ob/ob mice, and n = 2 for monkeys.

The PK properties of CHO-produced PF-06645849 were assessed in cynomolgus monkeys and rats following single IV or SC administration. The serum drug concentration was determined using a ligand-binding assay with a capture antibody that only cross-reacts with FGF21 protein with an intact C-terminus^20^. The mean IV and SC PK profiles are shown in Fig. 7 and key PK parameters were summarized in Table 1. The systemic clearance (Cl) of PF-06645849 was estimated to be 1.46 and 0.243 mL/hr/kg, IV terminal t_1/2_ was 72.9 and 199 hours, and SC bioavailability was ~50% and ~60% in rats (at 10 mg/kg) and monkeys (at 1 mg/kg), respectively. The time at which the maximum serum concentration was observed (T_max_) following a single 10 or 1 mg/kg SC dose was 48 and 72 hr post-dose in rats and monkeys, respectively.

**Table 1:** Summary of Key IV and SC PK Parameters for PF-06645849 in Rats and Monkeys.

Species	Dose (mg/kg)	Route	C_max_ (μg/mL)	T_max_ (h)	AUC_inf_ (μg*hr/mL)	Cl (hr/kg)	V_ss_ (mL/kg)	t_1/2_ (h)	F (%)
Rat	10	IV	178 ± 8.17	—	6850 ± 419	1.46 ± 0.087	111 ± 24.4	72.9 ± 7.45	—
	10	SC	23.3 ± 8.00	48 ± 0.0	3380 ± 543	—	—	42.3 ± 18.4	49.4 ± 8.00
Monkey	1	IV	29.8 ± 3.36	—	4140 ± 318	0.243 ± 0.019	62.5 ± 3.54	199 ± 14.1	—
	1	SC	7.21 ± 0.014	72.0 ± 67.9	2540 ± 7.07	—	—	140 ± 17.7	61.5 ± 4.90

C_max_, maximum observed concentration; T_max_, time to C_max_; AUC_inf_, area under the concentration-time curve from time zero extrapolated to infinity; Cl, systemic clearance; V_ss_, volume of distribution at steady state; t_1/2,_ terminal half-life; F (%), SC bioavailability. All values are expressed as (Means ± SD) with n = 3 for rats and n = 2 for monkeys.

### PD effects

**Figure 8:**
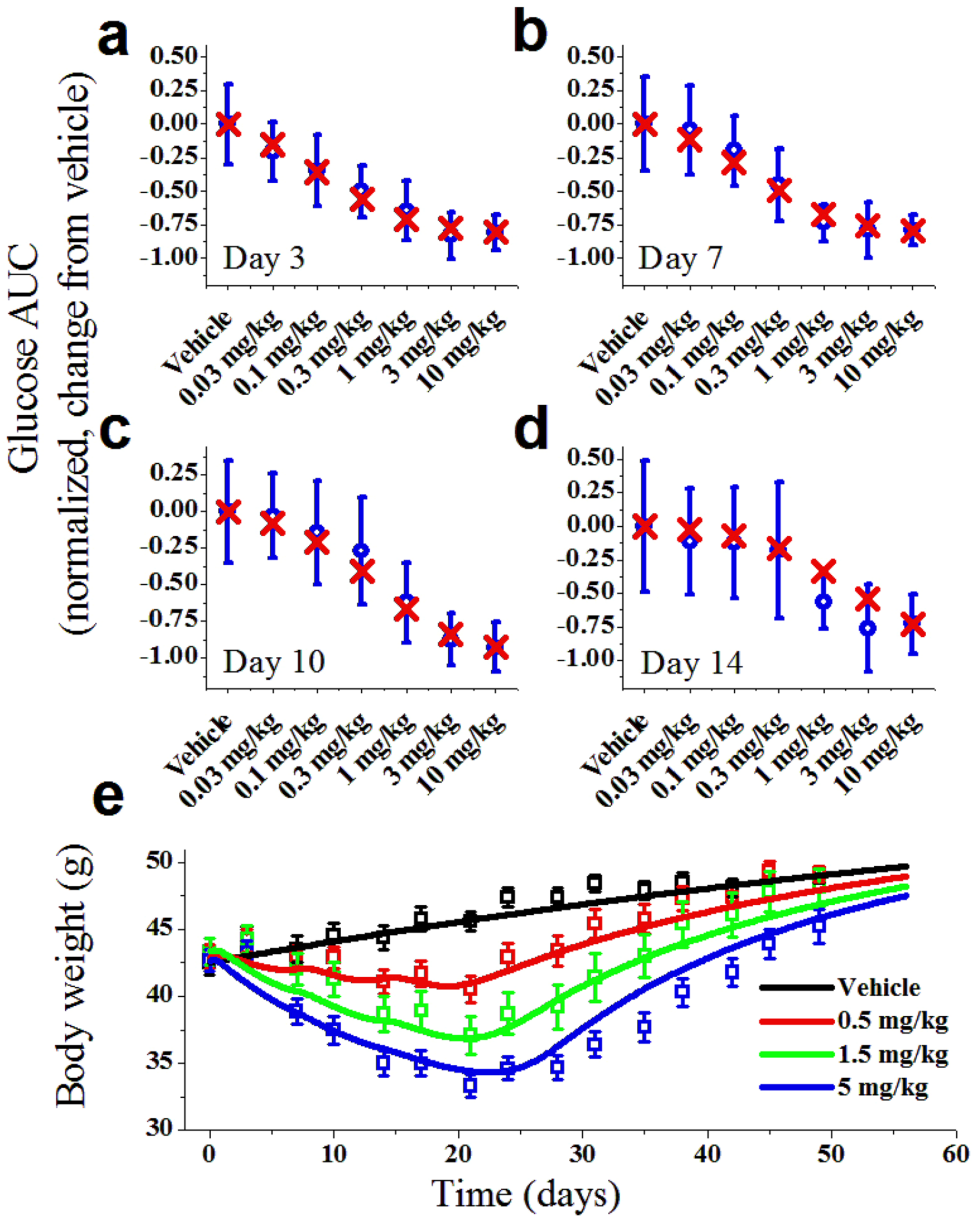
Integrated PKPD modeling of OGTT response in ob/ob mice (**a**–**d**) and BW loss in DIO mice (**e**). (**a**–**d**) Observed glucose AUC data (blue) vs. model prediction (red) on 3, 7, 10, and 14 days post a single SC injection of PF-06645849. Observed data are Mean ± SE with n = 12. (**e**) Body weight of DIO mice following 3 weekly SC doses of PF-06645849 (squares = observed data; curves = model prediction). Observed data are Mean ± E with n = 10.

To guide a dose selection of PD study, the PK of PF-06645849 was evaluated in ob/ob mice following a single dose at 0.1 to 10 mg/kg (Fig. 7c). The effect of PF-06645849 on glucose tolerance was then tested following a single SC administration in the range of 0.3 to 10 mg/kg. Consistent with previous reports on other FGF21 analogues, PF-06645849 robustly decreased glucose excursion in a dose-dependent manner, and the effects sustained at least for 2 weeks post dose (Fig. 8a–d). The maximal glucose AUC lowering observed in this study was similar to what has been reported previously^40,41^. PF-06645849 was also shown to decrease serum TG in ob/ob mice in a dose dependent manner and reached a similar maximal effect as observed for previous FGF21 analogues^9,40^ (Supplemental Fig. S6).

To assess the effect of PF-06645849 on BW loss, PF-06645849 was administered by weekly SC for three weeks at doses of 0.5, 1.5, and 5 mg/kg, respectively, in DIO mice, and BW was monitored twice weekly. Consistent with our previous reports on PF-05231023^41^, pharmacological administration of PF-06645849 decreased BW in DIO mice in a dose-dependent manner (Fig. 8e). Weight loss was apparent by day 7 in the 1.5 mg and 5 mg/kg groups, and by day 10 all dosed groups had substantial BW reduction compared to control. Weight loss was maximal at 7 days after the third dose (also the last dose, on day 14), after which BW rebounded and all dose groups achieved the same weight as control mice at the end of the study. The 0.5 mg/kg group caught up to control mice by day 45, whereas the 1.5 and 5 mg/kg groups caught up to control mice by day 70. We continued monitoring the BW of control and the 5 mg/kg group animals from day 70 to day 91 and interestingly, both groups gained weight at the same rate. Unlike the faster weight rebound after the last dose observed with our clinical molecule PF-05231023, the wash-out period in the animals show prolonged PD effects following the last dose that is consistent with a slower clearance rate for the current lead PF-06645849 and hence longer target engagement.

### PK/PD modeling

Finally, systems PKPD modeling was used to assess the integrated effect of loss of *in vitro* potency and prolonged PK of PF-06645849. The baseline normalized, placebo subtracted OGTT data in ob/ob mice and modeling results are shown in Fig. 8a–d. The model structure was the same as the one used previously to assess PD effect of PF-05231023^41^. A single fitted parameter, Kd, was sufficient to reproduce both the time and dose dependency of PF-06645849 on OGTT. The model estimated Kd value of 10 nM was in good agreement with the measured human *in vitro* potency of 16 nM (Fig. 4d).

Similarly, body weight change in DIO mice were assessed using the same model structure as described for PF-05231023 (Fig. 8e). Again, a single fitted parameter, Kd, was sufficient to reproduce both the time and dose dependency of PF-06645849 on BW loss. The model estimated Kd value in this case was 5 nM, which was also within the range of pERK assay potency observed in hASC.

Based on these analyses, our conclusion is that the molecular mechanism of PF-06645849 is similar to that of PF-05231023 and WT FGF21^41^, as similar receptor occupancy (RO) seems to translate into similar pharmacological effect. Therefore, an efficacious human dose could be projected to cover the same RO as either PF-05231023^21^ or LY2405319^12^, based on their similar mode of action. To simplify the analysis and to be more objective, we choose to match the RO of LY2405319 as it caused higher magnitude of increase in serum adiponectin compared to PF-05231023. Based on previous experience with PF-05231023, both rat and monkey single species scaling seems to predict human CT and NT clearance with reasonable accuracy (20); therefore, as described in methods both species were used for human PK prediction of PF-06645849. The human systemic clearance of PF-06645849 was estimated to be 0.12 to 0.35 mL/hr/kg, based on single species scaling of rat and monkey Cl, respectively, with a fixed exponent of 0.8^42,43^. The human PK profile was predicted using a 2-compartment PK model with Vc = 0.04 L/kg (central distribution volume), K_12_ = 0.0089 hr^−1^ (first-order distribution rate from central compartment to peripheral compartment), and K_21_ = 0.011 hr^−1^ (first-order distribution rate from peripheral compartment to central compartment). These values are derived based on analysis of PK parameters reported for typical human IgG1^44^. The SC absorption rate was set to be 0.011/hr, same as reported mean values for typical human IgG1^45^. SC bioavailability was set to be 50%, which is at the lower end of bioavailability observed in preclinical species (Table 1) and in line with reported values^45^. Terminal t_1/2_ is estimated to be either 9 days (using Cl scaled from rats) or 23 days (using Cl scaled from monkeys) based on these parameters.

An efficacious dose of PF-06645849 was then estimated by scaling the average clinical exposure of LY2405319 giving maximal weight loss^12^ by the relative potency between PF-06645849 and LY2405319 (as described in Experimental Procedures) and using the projected human pharmacokinetic parameters described above. We projected that a weekly SC doses of PF-06645849 range from 38 mg (assuming Cl of 0.12 mL/hr/kg as scaled from monkeys) to 111 mg (Cl of 0.35 mL/hr/kg, scaled from rat) would provide similar efficacy as daily SC dose of 10 mg of LY2405319.

## Discussions

Glycoengineering has been applied previously on therapeutic proteins to improve circulating half-life, *in vivo* activity, stability and solubility. Half-life extension of Aranesp^®^ relied solely on the introduction of two additional N-linked glycans. The overall carbohydrate content and isoform structures of this engineered EPO molecule was shown to play critical roles in modulating its serum half-life and *in vivo* biological activities. Isolation of lower pI and higher sialic acid content species during downstream manufacturing of this molecule was essential to achieve higher therapeutic potency^46^. Native FGF21 protein shares many similarities with EPO, such as its small molecular size susceptive of renal filtration, and short intrinsic half-life^47,48^. However, the enrichment approach used to produce Aranesp® would represent a substantial development and regulatory hurdle for targets such as FGF21 where dosing at 10 s to 100 s milligram range would likely be necessary to achieve efficacy, in contrast to a typical microgram dosage for rhEPO and Aranesp^®^. In the current work, we decided to apply a combination approach of Fc fusion in the context of glycoengineering, leveraging FcRn based half-life extension, platform CHO cell line production, and a robust protein A based manufacturing scheme.

A rationally designed FGF21[N171] glycan variant was selected amongst a number of engineered N-linked glycosylation variants that resulted in the elimination of key protease site between Pro171 and Ser172, and demonstrated prolonged serum stability both *in vitro* and *in vivo*. The resulting Fc-FGF21[N171] glycovariant has an apparent lower clearance and longer circulating half-life when compared to a previous point mutation variant using P171G to prevent protease cleavage at the same site (Fig. 2c,d)^15^, suggesting that in addition to disrupting a protease site by altering sequence, the presence of bulky carbohydrate moieties might be providing additional protection against serum protease cleavage, possibly through steric hindrance^28^.

CHO cell based expression system has long been the preferred, routine production system for biopharmaceutical industry, providing a robust and well-established manufacturing platform for monoclonal antibodies, Fc fusions, and native proteins, with key product attributes such as glycan compositions and other post translational modifications closely mimic those found in native human proteins. The discovery of a CHO cell specific proteolysis at position R19 of FGF21 initially came as a surprise (Fig. 3). Literature survey revealed that prior FGF21 analogues were either produced in yeast^17^, or bacteria system as an aglycosylated Fc fusion^15^. It is unclear if CHO cell specific proteolysis prevented its use for those analogues. In our study, the introduction of R19V mutation substantially improved conformational stability of the Fc-FGF21 fusion and manufacturability (Figs 3 and 4). To our knowledge, this represents the first time an FGF21 analogue can be produced successfully at large scale using the CHO expression platform.

In a standard physiological pH formulation at ambient temperature, aggregate formation for PF-06645849 at concentrations up to 60 mg/mL remained <5% over a 4-week study, and viscosity was <10 cP up to 100 mg/mL (Fig. 6). This pharmaceutical profile represents a substantial improvement over WT FGF21, as well as other FGF21 analogues that required less ideal SC formulation for maximal solution stability such as low pH and/or reduced protein concentration (patent # US 8722622 B2).

Compared to the HEK-derived Fc-FGF21[N171], the systemic clearance of CHO-derived PF-06645849 was further reduced from 2.51 to 1.46 mL/hr/kg in rats; and terminal t_1/2_ increased from ~33 hours to ~76 hours (Figs 2c and d vs. 7 and Table 1). This improvement could come from increased sialylation at the engineered glycan for CHO vs. HEK-derived proteins^31,49–51^, and minimal high mannose containing CHO glycan (Fig. 4c). It is worth noting that, while the systemic clearance of PF-06645849 in rats is substantially improved compared to either Fc-WT FGF21 or Fc-FGF21[P171G] (Fig. 2); it is still ~3x fold faster than a typical monoclonal antibody, suggesting contribution of residual protease-mediated degradation in this species. In monkeys, the Cl of PF-06645849 is 5–6x lower and terminal t_1/2_ is 3~6x longer than those of Fc-FGF21 variants engineered with other point mutations^15,52^. In fact, the Cl and t_1/2_ of PF-06645849 in monkeys are similar to those of a typical antibody^4^, suggesting minimal residual protease-mediated degradation for our glycoengineered variant in NHP. The species-difference in protease-mediated clearances of FGF21 was also observed for previous analogue PF-05231023^20^. In ob/ob mice, the serum drug concentrations at trough increased dose-proportionally from 0.1 to 10 mg/kg and it is within the therapeutic efficacious doses (Fig. 7c). Therefore, we concluded no evidence for meaningful contribution of target-mediated disposition for PF-06645849.

Efficacy studies with weekly SC dosing of PF-06645849 showed robust and sustained body weight loss in DIO mice, as well as profound improvements in OGTT and systemic TG lowering in ob/ob mice (Fig. 8 and Supplemental Fig. S6). Systems PK/PD modeling of body weight loss in DIO mice and OGTT improvement in ob/ob mice supports the hypothesis that the enhanced *in vivo* efficacy of PF-06645849 is primarily driven by improved PK properties. Taken together, the PK and PD profile of PF-06645849 makes it an attractive clinical asset for treatment of obesity and type 2 diabetes.

A reduced maximal effect (E_max_) in pERK phosphorylation assay was noted for Fc-FGF21 fusion proteins as compared to the WT FGF21 (Figs 2a and 4d), including both Fc-WT FGF21 and PF-06645849. This is likely due to the presence of Fc at the N-terminus of FGF21 impacting its interaction with FGFR1c, as this is the part of the protein that has been demonstrated to be the key determinant for *in vitro* E_max_ in pERK phosphorylation assays^26,27^. PF-05231023, which is generated by conjugation through a single point mutation A129C^16^ and thus retains free C- or N-terminus of the WT FGF21, showed the same EC_50_ and E_max_ as WT FGF21(Fig. 4d). It is worth noting that the reduced *in vitro* E_max_ does not seem to translate to reduced *in vivo* maximal efficacies, as treatment of PF-06645849 caused similar maximal effects in TG and OGTT-induced glucose AUC lowering as those following treatment of PF-05231023 in ob/ob mice (Fig. S6, Fig. 7a–d), and it caused even more body weight loss in DIO mice than those caused by PF-05231023 treatment (Fig. 7e)^41^.

Several long-acting FGF21 analogs have been reported that demonstrated robust efficacy in preclinical models and in the clinic^12,21,53^, including a very recent report of an engineered Fc-FGF21 variant^15,54^. In addition, agonist antibodies targeting components of the FGF21 signaling pathway such as KLB/FGFR1c^4,55^, or FGFR1c^56^, have also shown similar efficacy in preclinical species and more recently in the clinic^57^. It would be interesting to compare the safety and efficacy profiles of this class of compounds in humans with those of FGF21 based analogues. For the management of chronic diseases such as T2D and obesity, a longer acting molecule would be preferred over daily injections. Despite of their robust weight loss and TG lowering in humans, neither PF-05231023 nor LY2405319 was amendable for a prolonged SC dosing regimen^12^. More recently, a pegylated FGF21 analogue (BMS-986036) was reported to cause liver fat reduction and improvement of other biomarkers in obese type 2 diabetic subjects; however, it also requires daily SC injection for optimal therapeutic effects^58^.

In summary, PF-06645849 offers a substantial improvement over our previously reported clinical molecule PF-05231023 in terms of demonstrating glucose lowering and sustained weight loss in mice. Its mAb-like half-life and favorable pharmaceutical properties makes it an attractive clinical candidate. We project that a weekly or biweekly SC injection of PF-06645849 at ~40–100 mg would be sufficient to achieve comparable efficacy as reported for daily SC injection of 10 mg of LY2405319. Further studies will be carried out to demonstrate efficacy and safety of this molecule in NHPs before taking it to the clinic. The unique approach we employed here combining glycoengineering and Fc fusion guided by early assessment of manufacturability and molecular properties to generate a well behaved, long acting FGF21 has the potential to be applied more broadly as a platform technology to enhance properties of other protein therapeutics.

## Methods

### Cloning and protein expression

The expression plasmids of his-tagged FGF21 was constructed in pcDNA3.1 vector as N-terminal hexa-histidine residues followed by WT FGF21. The expression plasmid of Fc-FGF21 fusion was constructed as N-terminal Fc fragment (with three alanine mutations to eliminate effector function^36,37^) followed by a linker ((GlyGlyGlyGlySer)_3_ or (Gly)_8_ and WT human FGF21. For his-tagged FGF21 the resulting PCR product was treated with In-Fusion cloning enhancer following by insertion into pcDNA3.1 that had been treated with BamH1 and Hind3. For the Fc-fusion protein the resulting PCR product was treated with In-Fusion cloning enhancer following by insertion into an internal expression vector.

For protein production in transient system, HEK293F cells were transiently transfected with the expression plasmids by using 293fectin reagent and grown in FreeStyle293 media according to the manufacturer’s protocol (Invitrogen). The conditioned medium was collected by centrifugation at 6 days post transfection, and the supernatant was filtered by 0.2 μm filters for subsequent purification. The expression was confirmed by western blot with either a monoclonal anti-human FGF21 antibody (Abcam) or a peroxidase conjugated mouse anti-human IgG antibody (Thermo Scientific).

For stable expressing cells, CHO cells were transfected with the expression plasmid by Lipofectamine 2000 (Invitrogen), according to the manufacturer’s instructions. Stable pools were selected with 1 mg/mL G418 and 50 nM methotrexate for 2 to 3 weeks. The conditioned medium was collected by centrifugation at 2,000 × g for 10 min after 8 days culture for stable expression. The supernatant was filtered by 0.2 μm filters and stored at 4 °C for subsequent purification. Expression level of protein was quantitated by protein A biosensors with Octet (ForteBio).

### Purification

His-tagged variants was purified on a HiTrap chelating column (GE healthcare) using a linear gradient from 10 to 250 mM imidazole in sodium phosphate buffer at pH 7.6. Transiently produced Fc fusions of FGF21 variants were purified using a RMP Protein A column (GE healthcare), eluted with 50 mM citric acid, pH 3.3, and neutralized with Tris-HCl (pH 8.0). Aggregated protein fractions were removed and buffer exchanged into PBS using a preparative Superdex 200 column (GE healthcare). Fc fusions of FGF21 from stable CHO cells were purified using the same RMP Protein A column. A subsequent Toyopearl Butyl 650 S resin (Tosoh) equilibrated with 1 M NaCl, 50 mM Tris pH 8.0 was used to isolate the intact Fc-FGF21 fusion in a FT step. The protein was exchanged into 20 mM Tris/HCl pH 8.0 and further purified on Q Sepharose FF resin (GE) with a load challenge of 25 g/L using a linear gradient from 25 to 250 mM NaCl in 20 mM Tris/HCl pH 8.0 over 4 CV. The monomeric status of all FGF21 proteins was confirmed by Superdex 200 10/300 GL (GE Healthcare) column with a flow rate of 0.5 ml/min PBS.

### Characterization of N-glycosylation variants of FGF21

The occupancy of the engineered N-link site was calculated using a Caliper LabChip GXII with a protein chip and Perkin Elmer reagents. *In vitro* serum stability test of FGF21 was carried out in either PBS, pH 7.2 or human serum (Sigma-Aldrich) for 96 hours at 37 °C with time points taken at 0, 48 and 96 hours. Clipping was monitored by Western blot analysis using the same anti-human FGF21 antibody (Abcam) as described above.

### Kinase phosphorylation assay for FGF21 variants

Human adipose stem cells (hASC) were purchased from Zenbio (lot# 062801) and grown up to 6 passages in growth media containing DMEM/F12 with 10% v/v FBS, 5 ng/ml hEGF (Invitrogen 13247–051), 1 ng/ml bFGF (Invitrogen 13256-029), and 0.25 ng/ml hTGF (Sigma T1654). The pERK1/2 assay was carried out as previously described^25^. All concentrations were converted to molarity using the full molecular weight of the individual molecules. Resulting data were normalized to the average signal obtained at the highest concentration of WT FGF21, which was included as positive control for all studies. EC_50_ and E_max_ were estimated using a 4-parameter variable-slope Hill function in GraphPad Prism (version 7.02, GraphPad Software Inc.)

### MS analysis

Protein samples were deglycosylated by PNGase F (New England BioLabs, Ipswich, MA) or IgGZero (Genovis, Cambridge MA) according to manufacturer’s recommendation and further reduced by TCEP (Thermo Fisher, Waltham, MA). The samples were acidified by diluting 1:1 with 0.1% formic acid (Sigma-Aldrich, St Louis, MO) followed by liquid chromatography mass spectrometry analysis (LC-MS). LC-MS analysis was performed using a Waters Xevo Q-TOF G2 mass spectrometer (Waters, Milford, MA) coupled to an Agilent (Santa Clara, CA) 1100 capillary HPLC. The deglycosylated and reduced samples were separated over an Agilent Poroshell 300SB-C8 (0.5 × 75 mm) column maintained at 80 °C with a flow rate of 20 µl/min. Mobile phase A was water with 2% acetonitrile and 0.1% formic acid, and mobile phase B was acetonitrile with 2% water and 0.1% formic acid. The mass spectrometer was run in positive MS only mode scanning from 800 to 2000 m/z and data was acquired with MassLynx (Waters) 4.1 software. The TOF-MS signal was summarized and deconvoluted using MaxEnt1 (Waters) program.

### *In vitro* assay of the Arg19-Tyr20 cleavage

CHO conditioned media of untransfected cells was harvested from a 7 day culture. Purified Arg19 variants of Fc-FGF21[N171] from HEK293F expression was diluted to a final concentration of 100 μg/mL into the CHO conditioned media and incubated at room temperature. Samples were taken at different time intervals and the progression of clipping was monitored by western blot using the same anti-human IgG antibody (Thermo Scientific) described above. All Western blot images were acquired through BioRad Chemi Doc XRS Western Imaging System, or Kodak 2000R Digital Imaging System, or Kodak BioMax film developed in an automated processor.

### Differential scanning calorimetry (DSC)

Thermal stabilities of Arg19 variants of Fc-FGF21[N171] were analyzed using the VP-DSC (MicroCal). Protein concentration was 0.02 mM in PBS, and sample and reference cells were heated from 10 °C to 100 °C at a scan rate of 100 °C per hour. The heat capacity difference between cells was corrected for any system heat and recorded and analyzed using Origin7.0 software from MicroCal.

### Formulation and stability

FGF21 proteins were buffer exchanged into 20 mM Tris, 8.5% sucrose at pH 7.5, and were concentrated using Amicon Ultra spin concentrators (Millipore). Protein concentrations were determined using a SOLO-VPE Spectrophotometer (C Technologies Inc). The buffered protein samples were incubated at 25 °C and 4 °C up to four weeks, with time points taken at day 0, 2, 7, 14, 28 days. At each time point, the incubated samples were analyzed on Agilent 1100 HPLC system with YMC-Pack Diol 200 (YMC) analytical size exclusion chromatography column and PBS supplemented to 400 mM NaCl as running buffer. Viscosity was determined using a MCR-302 rheometer (Anton Paar) with a CP25 measuring system. The data was analyzed using the Rheoplus V 3.62 software.

### Animal studies

All animal studies were conducted in accordance with animal care and use protocols approved by the Institutional Animal Care and Use Committee (IACUC) of Pfizer, Inc. All experiments within this manuscript were undertaken to minimize animal suffering during the experiment.

### PK studies

PK of HEK-derived Fc-FGF21, Fc-FGF21[P171G], and Fc-FGF21[N171] was evaluated in male rats at 10 mg/kg following a single IV administration. PK of PF-06645849 (CHO-derived) was evaluated in male rats and cynomolgus monkeys at 10 and 1 mg/kg following a single IV or SC injections, respectively. Serum samples were collected at predose, 0.25, 1, 2, 6, 24, 48, 72, 96, 168, 240, 336, and 501 (monkeys only) hours post-dose, and stored at −80 °C until used for analysis. Serum levels of PF-06645849 are determined using an in-house Meso Scale Discovery^®^ (MSD) ligand-binding assay. PF-06645849 is captured by an anti-FGF21 C-terminus-specific antibody^20,41^ with functional C-terminus, and detected with a mouse anti-human IgG Fc antibody (Southern Biotech, Birmingham, AL) labeled in-house with ruthenium at a 6:1 ruthenium to antibody molar coupling ratio. All pharmacokinetic parameters were determined from individual animal data using non-compartmental analysis using software WinNonlin (Version 5.2, Pharsight, CA).

### PD studies

OGTT and weight loss studies were performed as reported^41^. For OGTT study, PF-06645849 was administered by SC injection at indicated doses to ob/ob mice, and OGTTs were conducted on day 3, 7, 10, and 14 post dose, respectively. Briefly, mice were fasted overnight, administered an oral gavage of 1 g/kg dextrose, and blood glucose concentrations were measured using handheld glucometers at 0, 15, 30, 60, and 120 min post glucose challenge. For the weight loss studies, DIO mice were administered PF-06645849 by SC injection at doses 0, 0.5, 1.5 and 5 mg/kg on study day 0, 7 and 14, and then subjected to washout. Body weight was measured twice a week until day 91.

### PK/PD modeling

PK/PD modeling was performed as described previously^41^ for both OGTT in ob/ob mice and body weight change in DIO mice, with the following changes. For OGTT modeling, a single process with measured SC t_1/2_ (44 hour) was used to describe the clearance of intact PF-06645849, and a single Kd was fit to optimize model agreement with the experimental data; all other model parameters were identical to the previous work describing a different FGF21 modality^41^. For BW modeling in DIO mice, the drug again was subject to a plasma clearance with measured t_1/2_, and the binding affinity was used as a fit parameter.

### Data availability

All data generated or analyzed during this study are included in this published article (and its Supplementary Information files).

## Electronic supplementary material

Supplementary Information
