# Supplementary material for: Glyco-engineered Long Acting FGF21 Variant with Optimal Pharmaceutical and Pharmacokinetic Properties to Enable Weekly to Twice Monthly Subcutaneous Dosing
Source: Sci Rep. 2018 Mar 9;8:4241. doi: 10.1038/s41598-018-22456-w (PMC5844872; doi:10.1038/s41598-018-22456-w)
Supplement: Supplementary file 1 — Supplementary Information [file 41598_2018_22456_MOESM1_ESM.pdf]

SUPPLEMENTARY INFORMATION:

Glyco-engineered Long Acting FGF21 Variant with Optimal Pharmaceutical and Pharmacokinetic Properties to Enable Weekly to Twice Monthly Subcutaneous Dosing

Yan Weng[1, ^], Tetsuya Ishino[1, ^], Annette Sievers [1], Saswata Talukdar[2], Jeffrey R. Chabot [1], Amy Tam [1], Weili Duan [1], Kelvin Kerns [1], Eric Sousa [1], Tao He [1], Alison Logan[2], Darwin Lee[2], Dongmei Li[2], Yingjiang Zhou[2], Barbara Bernardo[2], Allison Joyce[1], Mania Kavosi[1], Denise M. O'Hara[1], Tracey Clark[1], Jie Guo[1], Craig Giragossian[1], Mark Stahl [1], Roberto A. Calle[2], Ron Kriz [1], Will Somers[1], Laura Lin [1]*

[1] BioMedicine Design, Pfizer Worldwide Research and Development, Cambridge, MA (Y. W., T. I., A. S., J. R. C., A. T., W. D., K. K., E. S., T. H., M. S., R. K., W. S., L. L.), Andover, MA (A. J., M. K., D. M. O.), Groton, CT (T. C., C. G), and La Jolla, CA (J. G.).

[2] Internal Medicine, Pfizer Worldwide Research and Development, Cambridge, MA (S.T., D. L., D. L., Y. Z., B. B., R. A. C.), and Groton, CT (A. L.)

[^] These authors contributed equally. * To whom correspondence should be addressed: Dr. Laura Lin, Tel: +1 (617) 6746462, Email: laura.lin@pfizer.com

Supplemental Methods

PK Analysis - All pharmacokinetic parameters were determined from individual animal data using non-compartmental analysis using software WinNonlin (Version 5.2, Pharsight, CA). These included maximum observed concentration in serum (C_{max}), time to C_{max} (T_{max}), area under the concentration-time curve from time zero extrapolated to infinity (AUC_{inf}), terminal half-life ($t_{1/2}$), systemic clearance (Cl), and volume of distribution at steady state (V_{ss}). AUC values were calculated using the log-linear trapezoidal rule; Cl was calculated as ((IV dose)/$AUC_{inf, IV}$); terminal $t_{1/2}$ was calculated as (ln(2)/(slope of the terminal log-linear phase)); SC bioavailability (F) is calculated as dose-normalized ($AUC_{inf, SC}$ /$AUC_{inf, IV}$)%.

Supplemental Figure Legend:

Figure S1. N-glycosylation engineering of FGF21. a. MS identification of P171 proteolysis in human plasma after 92 hours incubation, additional lower abundance sites were also observed; b. MOE model of FGF21; C, D, and E, introduced N-link sites in the N-terminal (d), middle (d), and C-terminal region (e) of FGF21.

Figure S2. Western blot analysis of serum stability of selected FGF21 variant following incubation in human serum for up to 96 hr (Full Western blot images for Figure 1c, dash line indicates different proteins on the same blot)

Figure S3. pERK phosphorylation assay of WT FGF21, WT FGF21-Fc, and Fc-WT FGF21. Values are Mean ± SD (n=3).

Figure S4. Western blot analysis of HEK-produced R19 mutants of Fc-FGF21[N171] following incubation in conditioned CHO cell media for 3 days (Full Western blot images for cropped pictures in Figure 3e)

Figure S5. SDS-PAGE of Fc-FGF21[R19V][N171] following ButylFF (GE) purification. lane 1 , 7, 10: Invitrogen Mark12 standard; lane 2: load; lane 3: flow-through; lane 4: one column volume wash ; lane 5: second wash; lane 6: elution; lane 8 and 9: separate butyl HIC purification showing one column volume was (8) and second wash (9) (full gel image for cropped picture figure 3f)

Figure S6. Serum TG AUC in ob/ob mice following a single SC administration of PF-06645849 at 0.03, 0.1, 0.3, 1, 3, and 10 mg/kg, respectively. AUC were calculated based on serum TG measurements collected at baseline and on days 3, 7, 10, and 14 post dose. Values presented are Mean ± SE with n=12. **/*** means statistically different as compared to PBS group (P-value < 0.05 or 0.01, 1 way ANOVA).

Supplemental Table S1. Characterization of N-glycosylation variants of FGF21: % unglycosylated and relative pERK activity

Mutants	% unglycosylated	EC_{50}, pERK, (fold change relative to wtFGF21)
wt FGF21	N/A	1
N5	52%	inactive
N6	< 1 %	70.8
N7	< 1 %	inactive
N8	< 1 %	inactive
N9	62%	inactive
N10	< 1 %	inactive
N11	< 1 %	15.7
N12	< 1 %	inactive
N13	< 1 %	85.3
N14	2%	31.0
N15	< 1 %	1221.6
N16	< 1 %	66.1
N17	< 1 %	inactive
N18	2%	119.4
N121	>80%	n/d
N125	>80%	n/d
N129	>80%	n/d
N155	< 1 %	84.3
N156	< 1 %	23.5
N157	< 1 %	556.2
N158	< 1 %	766.6
N160	35%	7006.2
N162	< 1 %	inactive
N163	< 1 %	inactive
N164	< 1 %	inactive
N165	80%	inactive
N166	7%	inactive
N167	3%	15.5
N168	1%	inactive
N169	4%	188.8

N170	29%	20.3
N172	11%	23.1
N174	3%	129.7
N175	< 1 %	95.9
N176	3%	69.8
N177	< 1 %	336.0
N178	99%	N/A
N179	15%	inactive

Supplemental Table S2. Relative R19 cleavage and hASC pERK activity of Fc-FGF21 Arg19 variants expressed in HEK cell lines

Arg19 Variants	Cleavage	EC_{50}, pERK (fold change relative to Fc-FGF21[N171])
Fc-FGF21[N171]	+++	1
Fc-FGF21[R19A][N171]	++	1.3
Fc-FGF21[R19Q][N171]	++	1.7
Fc-FGF21[R19E][N171]	+++	6.5
Fc-FGF21[R19Y][N171]	++	0.6
Fc-FGF21[R19W][N171]	++	1.0
Fc-FGF21[R19F][N171]	++	0.4
Fc-FGF21[R19I][N171]	-	1.5
Fc-FGF21[R19L][N171]	-	0.8
Fc-FGF21[R19V][N171]	-	0.9
Fc-FGF21[R19K][N171]	+++	1.2
Fc-FGF21[R19M][N171]	-	1.1

Figure S1.

a

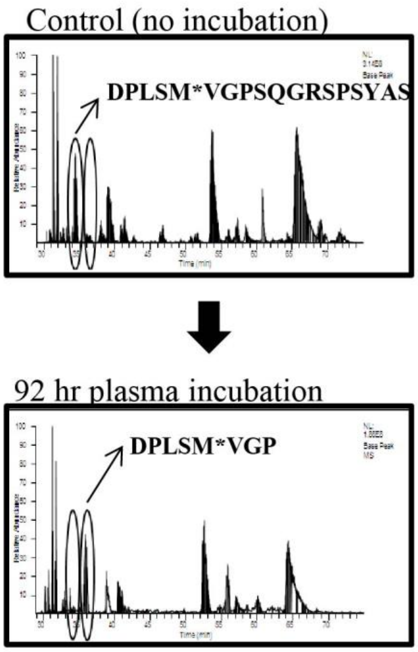

b

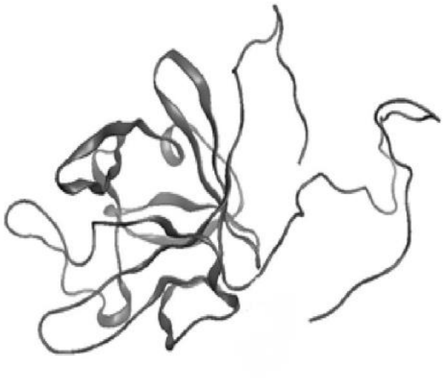

c

```
2    PIPDSSPLLQFGGQVRQRYL 21

N4   PINGSSPLLQFGGQVRQRYL (2 mutations)
N5   PIPNSSGLLQFGGQVRQRYL (2 mutations)
N6   PIPDNSTLLQFGGQVRQRYL (2 mutations)
N7   PIPDSNGTLQFGGQVRQRYL (3 mutations)
N8   PIPDSSNLTQFGGQVRQRYL (2 mutations)
N9   PIPDSSPNLTFGGQVRQRYL (2 mutations)
N10  PIPDSSPLNQTGGQVRQRYL (2 mutations)
N11  PIPDSSPLLNFTGQVRQRYL (2 mutations)
N12  PIPDSSPLLQNGTQVRQRYL (2 mutations)
N13  PIPDSSPLLQFNGTVRQRYL (2 mutations)
N14  PIPDSSPLLQFGNQTRQRYL (2 mutations)
N15  PIPDSSPLLQFGGNVTQRYL (2 mutations)
N16  PIPDSSPLLQFGGQNRTRYL (2 mutations)
N17  PIPDSSPLLQFGGQVNQTYL (2 mutations)
N18  PIPDSSPLLQFGGQVRNRTL (2 mutations)
```

d

```
121   NKSPHRDPAPRG 132

N121  NKSGHRDPAPRG (1 mutations)
N125  NKSPNRTGAPRG (3 mutations)
N129  NKSPHRDPNGTG (3 mutations)
```

e

```
154   APQPPDVGSSDPLSMVGPSQGRSPSYAS 181
N155  ANQTGDVGSSDPLSMVGPSQGRSPSYAS (3 mutations)
N156  APNGTDVGSSDPLSMVGPSQGRSPSYAS (3 mutations)
N157  APQNGTVGSSDPLSMVGPSQGRSPSYAS (3 mutations)
N158  APQPNGTGSSDPLSMVGPSQGRSPSYAS (3 mutations)
N159  APQPPNVTSSDPLSMVGPSQGRSPSYAS (2 mutations)
N160  APQPPDNGSSDPLSMVGPSQGRSPSYAS (1 mutations)
N161  APQPPDVNSSDPLSMVGPSQGRSPSYAS (1 mutations)
N162  APQPPDVGNSTGLSMVGPSQGRSPSYAS (3 mutations)
N163  APQPPDVGSNGTLSMVGPSQGRSPSYAS (3 mutations)
N164  APQPPDVGSSNGTSMVGPSQGRSPSYAS (3 mutations)
N165  APQPPDVGSSDNLSMVGPSQGRSPSYAS (1 mutations)
N166  APQPPDVGSSDPNSTVGPSQGRSPSYAS (2 mutations)
N167  APQPPDVGSSDPLNMTGPSQGRSPSYAS (2 mutations)
N168  APQPPDVGSSDPLSNVTGSQGRSPSYAS (3 mutations)
N169  APQPPDVGSSDPLSMNGTSQGRSPSYAS (2 mutations)
N170  APQPPDVGSSDPLSMVNGSQGRSPSYAS (2 mutations)
N171  APQPPDVGSSDPLSMVGNSTGRSPSYAS (2 mutations)
N172  APQPPDVGSSDPLSMVGPNQTRSPSYAS (2 mutations)
N173  APQPPDVGSSDPLSMVGPSNGTSPSYAS (2 mutations)
N174  APQPPDVGSSDPLSMVGPSQNRSGSYAS (2 mutations)
N175  APQPPDVGSSDPLSMVGPSQGNSTSYAS (2 mutations)
N176  APQPPDVGSSDPLSMVGPSQGRNGSYAS (2 mutations)
N177  APQPPDVGSSDPLSMVGPSQGRSNSTAS (2 mutations)
N178  APQPPDVGSSDPLSMVGPSQGRSPNYTS (2 mutations)
N179  APQPPDVGSSDPLSMVGPSQGRSPSNAS (1 mutations)
```

Figure S2.

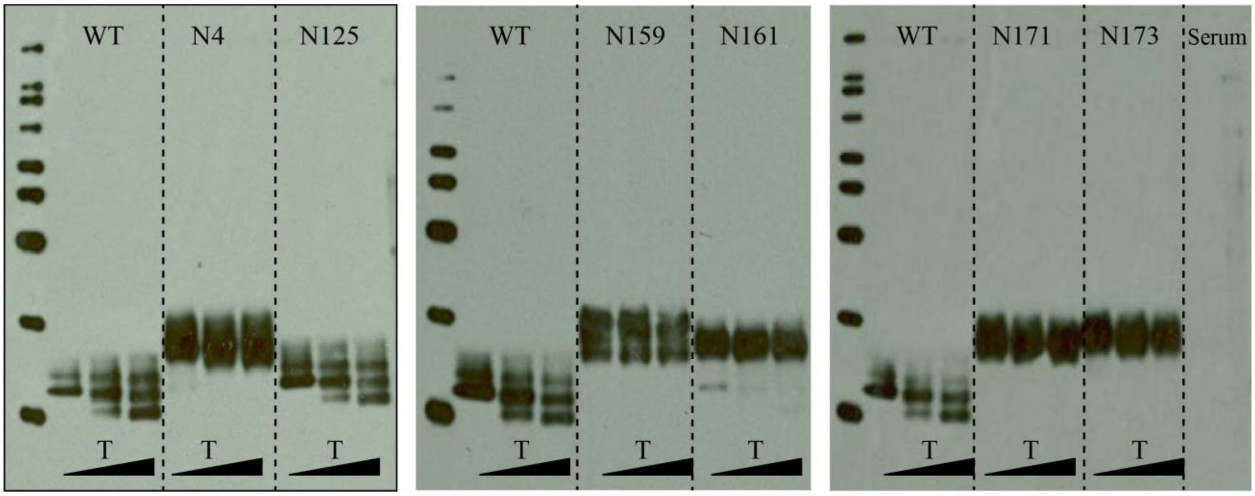

Figure S3.

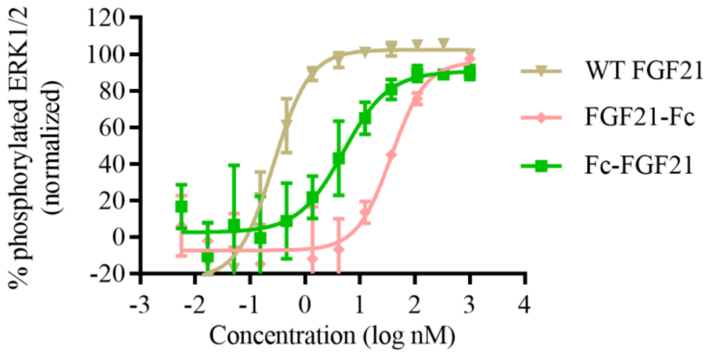
% phosphorylated ERK1/2 (normalized)
120
100
80
60
40
20
0
-20
-3
-2
-1
0
1
2
3
4
Concentration (log nM)
WT FGF21
FGF21-Fc
Fc-FGF21

Figure. S4.

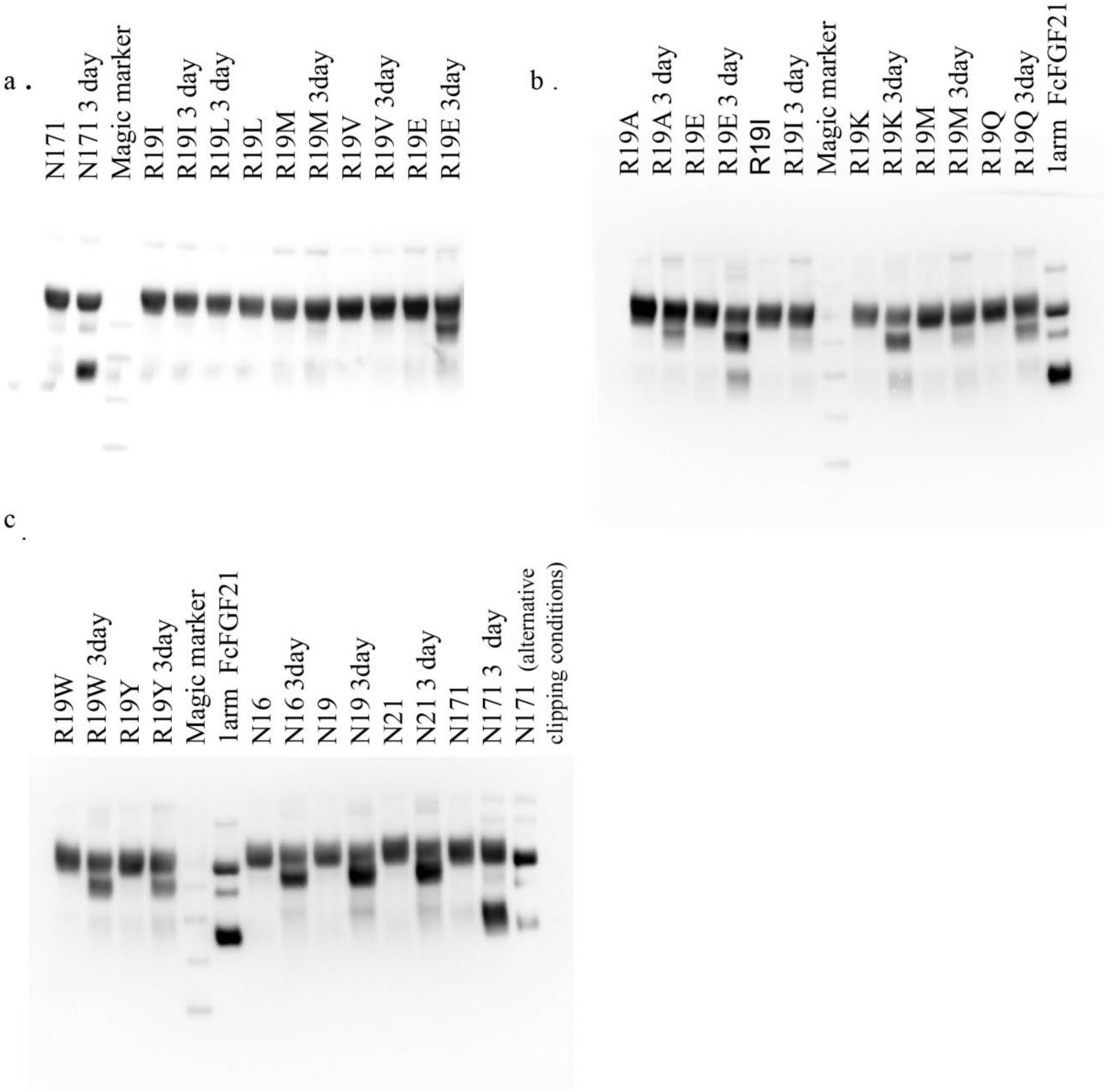
a.
N171
N171 3 day
Magic marker
R19I
R19I 3 day
R19L 3 day
R19L
R19M
R19M 3day
R19V
R19V 3day
R19E
R19E 3day
b.
R19A
R19A 3 day
R19E
R19E 3 day
R19I
R19I 3 day
Magic marker
R19K
R19K 3day
R19M
R19M 3day
R19Q
R19Q 3day
1arm FcFGF21
c.
R19W
R19W 3day
R19Y
R19Y 3day
Magic marker
1arm FcFGF21
N16
N16 3day
N19
N19 3day
N21
N21 3 day
N171
N171 3 day
N171 (alternative clipping conditions)

Figure S5.

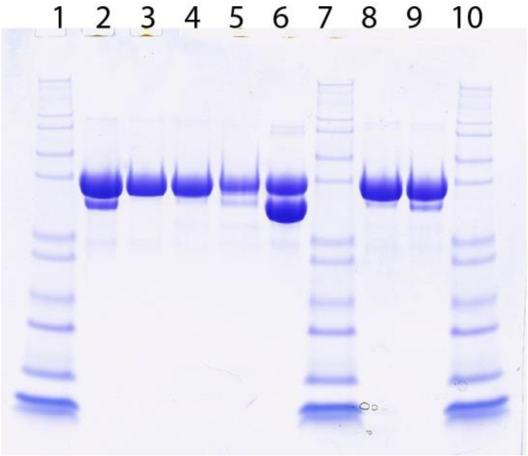

Figure S6.

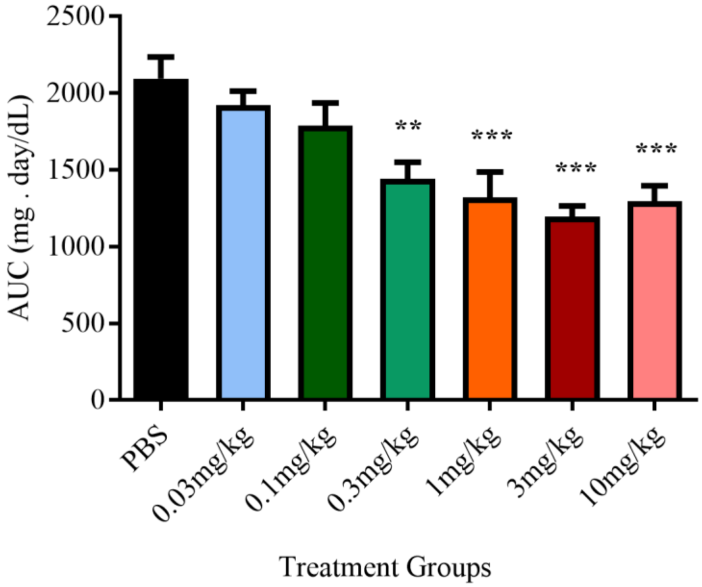

2500
2000
1500
1000
500
0
AUC (mg . day/dL)
**
***
***
***
PBS
0.03mg/kg
0.1mg/kg
0.3mg/kg
1mg/kg
3mg/kg
10mg/kg
Treatment Groups
